# The 2010 Gulf of Mexico Oil Well Blowout: A Little Hindsight

**DOI:** 10.1371/journal.pbio.1001049

**Published:** 2011-04-19

**Authors:** Carl Safina

**Affiliations:** Blue Ocean Institute, School of Marine and Atmospheric Sciences, Stony Brook University, Stony Brook, New York, United States of America

**Figure pbio-1001049-g001:**
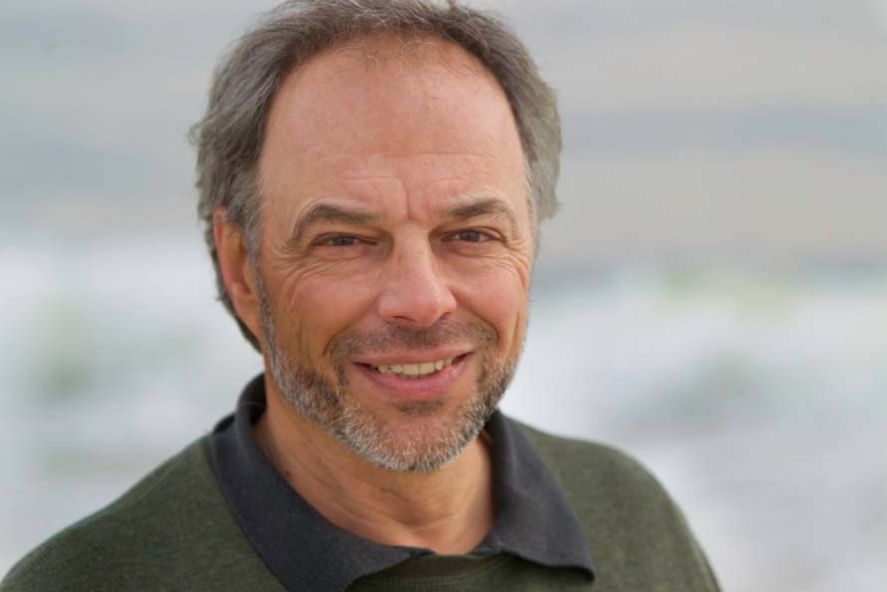
About the Author. Carl Safina holds a PhD in ecology from Rutgers University and has published over 150 research papers, book chapters, books, and articles on seabird ecology, fisheries, and environmental policy. He is founding president of Blue Ocean Institute and adjunct professor in marine science and science communication at Stony Brook University. He has studied the ocean as a scientist, stood for it as an advocate, and conveyed his travels among sea creatures and fishing people in lyrical nonfiction writing. His first book, *Song for the Blue Ocean*, was chosen as a *New York Times* Notable Book of the Year, a *Los Angeles Times* *Best Nonfiction selection, and a Library Journal* Best Science Book selection; it won the Lannan Literary Award for nonfiction and a MacArthur “genius” prize. Dr. Safina’s second book, *Eye of the Albatross*, won the John Burroughs Medal for the year’s best book about the natural world and was chosen by the National Academies of Science, Engineering and Medicine as the “Year’s Best Book for Communicating Science.” The *New York Times* chose his *Voyage of the Turtle* as an Editor’s Choice. His first children’s book was published in 2010. *The View from Lazy Point* recently won him a special Guggenheim Fellowship and was also selected a *New York Times* Editor’s Choice. His book about the oil blowout in the Gulf of Mexico is *A Sea in Flames* (Crown Publishers, 2011). Dr. Safina hosts *Saving the Ocean* on PBS television.

[Fig pbio-1001049-g001]No profile of events can capture all the facts, the chaos, and the many thousands of pages devoted to what the Gulf of Mexico oil blowout was—and was not.

During several visits to the Gulf region in 2010 and in the months I spent writing a book on the subject, the best way I found to make sense of the blowout's many facets was as conceptual topography, its contours shaped by the interlaced factual and emotional features of the event. Perceptions were important drivers of the effects of the event. Economic effects largely reflected perceptions by tourists and seafood consumers, and psychological effects resulted from deep uncertainty over ecological effects and consequently the future viability of fishing and tourism. It seemed an event unfolded in three acts: First, the factors leading up to the blowout. Two, the varied responses during the blowout while oil was still streaming from the well. Third, the post-leak period when assessment, study, and comparison merged the technological, political, emotional, and scientific components that comprised the event [1].

It is tempting to jump from the blowout to a discussion of America's energy needs and the world's energy future. Those larger implications can seem to be the main messages of the blowout. But those messages exist independent of the blowout. That's why I will resist that connection until we discuss the blowout itself.

## The Blowout Itself

In 2008, the multinational energy company BP leased a piece of seafloor in the Gulf of Mexico about 80 km (50 miles) from Louisiana's southern shore. The plot was named Macondo after a fictional town hewn from a “paradise of dampness and silence” in Gabriel García Márquez's novel *One Hundred Years of Solitude*. To do the drilling, BP hired the global drilling company Transocean and its drilling rig Deepwater Horizon. The rig itself was nearly 122 m (400 ft) tall, its drilling platform bigger than a football field.

Deep-water drilling is relatively new. In just the last decade, the number of wells in water deeper than a mile has gone from only two dozen to nearly 300. Increasing complexity increases risks; minimizing challenges in order to successfully overcome them creates a tendency to downplay risks. But as drilling technology advanced rapidly, accident preparedness and problem-response technology—and United States law—remained stuck in the past, modeled on an *Exxon Valdez*–type tanker leak.

Though BP owned the lease, contractors like Halliburton, which did the cementing jobs that held the well's liners in place, and M-I SWACO, which dealt with the continually circulated drilling fluids, did almost all of the work.

The distance from the rig to the sea floor was just under 1.6 km (1 mi). Sea floor to the bottom of the well: just over 4 km (about 13,368 ft, or 2.5 mi). A total of 5.6 km (18,360 ft) from sea surface to well bottom (>3.5 mi). Humans cannot dive to such depths, so all the work is done remotely.

Though two-and-a-half miles long, the well's top was just 1 meter across, its bottom merely 172 mm (7 in). As the well gets dug, engineers line its sides with steel casing assembled at the surface and sent down the well. Some sections reach ≈600 m (2,000 ft) long. As drilling continues and the well deepens, narrower sections are slid through existing sections and cemented into place. Sitting atop the well is the failsafe, the blowout preventer, a 12 m (40 ft) high stack of valves that can squeeze or cut and seal the drill pipe if upward-gushing oil and gas threaten to become uncontrollable.

Various problems had put the job behind schedule and over budget. In late April 2010, having discovered a commercially valuable reservoir—drilling rigs are meant to discover, not extract, oil and gas—drillers prepared to seal the well and withdraw, facilitating later extraction of the petroleum.

Several artificial drilling fluids called “mud” are constantly circulated between the rig and the bottom of the well and back. These heavier-than-water fluids perform several functions, including bringing up rock loosened by the drill bit, facilitating examination of materials and hydrocarbons found, and importantly, keeping a miles-high stack of weight to counter the upward pressure of any hydrocarbons coming out of the well walls in the petroleum-bearing zone. The fluids are the stopper in the pressurized bottle. They are expensive; the volume required in a deep-water well can cost more than half a million dollars.

The Macondo well's problems included loose walls, through which much pressurized drilling fluid escaped. Engineers dealt with this by mixing a special batch of viscous fluid designed to seal the porosity in a way analogous to sealing a leak in a flat tire with a spray. They had mixed more than they used, and were faced with a disposal problem. Bringing the material back to land for disposal would have required the expense of transporting it and handling it as hazardous waste. But drilling rules allowed them to mix it with other drilling fluid and send it down the well. Rig workers sometimes use a different fluid to help them mark or separate the border between two kinds of fluids. When they see such a “spacer” return to the rig, they know they are between two different fluids.

On the day of the blowout, the main tasks were: pump a cement plug hundreds of feet high into the well to seal the hydrocarbons in, and recapture the drilling fluid and displace it with much lighter seawater.

The cements used are not normal concrete but special mixtures designed to deal with pressure and the heat inside so deep a well (above the boiling point of water). To see whether the cement job succeeded, the rig team lessened pressure on the well by displacing some of the heavy drilling fluid with seawater. Between the fluid and water they used as a spacer the viscous fluid they wanted to dispose of—doing so was highly unusual.

The test of the cement was to reduce pressure from above, then make certain that no pressure was building in the well from below. The test protocol called for a pressure gauge reading of zero on a particular pipeline to the rig. And that line showed zero pressure.

But on a different line, another gauge was showing pressure building. The gauge indicating building pressure was correct. The line showing zero pressure was clogged with the viscous spacer. The increasing pressure indicated that the cement had failed, and that pressurized oil and gas were entering the well. But rig workers convinced themselves that the gauge showing zero pressure was correct and the other was an anomaly.

Because they intended to discard the spacer, when they returned it up to the rig they shunted it overboard, thereby temporarily bypassing other pressure gauges that could have provided further warning.

When they realized they had a problem, confusion and issues over authority delayed assessment of its severity and caused hesitation in initiating attempts to activate the blowout preventer or disconnect the rig from the mile-long pipe to the seafloor. They had re-routed the fluid return back onto the rig when large amounts of methane reached the surface. Generator turbines sucked the gas in, causing ignition.

One worker recognized the need to shut down the generators but knew he was not authorized to do so, and consequently did not. The rig's chief electrician has asserted that inhibited audio alarms also inhibited computer-activated emergency shutdown of air vents and power.

The subsequent explosions killed 11 people. They also damaged controls to the blowout preventer and the emergency disconnect system, rendering them unresponsive. Connected to a nearly infinite source of fuel, the rig became an inferno. More than 100 other people escaped in lifeboats or by leaping into the sea. The rig burned for two days, then sank on April 22, 2010. The broken pipe at the seafloor continued spewing oil until, after several attempts to cap or clog the well, a new cap succeeded in mid-July.

To review: the cement mixture failed, highly irregular spacer material clogged a key pressure gauge, crew members failed to correctly interpret disparity between pressure gauges, other gauges were bypassed or overlooked at critical times, inhibited alarms may have prevented automatic shutdown of the ignition source, and hesitation over authority prevented manual shutdown that might have averted ignition of the pressurized hydrocarbons.

A lot of what went wrong involved matters of judgment. Even the cement failure appears at least partly linked to inadequate testing, as well as to formulation and the difficulties caused by depth. Perhaps the main lesson is: even with literally billions of dollars and many lives at stake, incentives to hurry and to show bravura can seem more important in the moment.

## Unpreparedness

BP had a federally approved Gulf of Mexico spill response plan that explained what it would do for walruses and sea lions—creatures that don't live in the Gulf of Mexico. Major sections were merely cut-and-pasted from Arctic plans. No one paid attention to them. In a region full of oil rigs and warehouses full of hardware, nowhere was there a device for shutting off a leaking pipe 1 mile deep. All the response equipment available was similar to what it had been in the 1970s: booms adequate to contain small spills inside harbors, and dispersant chemicals.

Quickly overcome by minor wind and wave action, the booms did little to contain the oil. About 2 million gallons of chemical dispersants were added to the oil at the surface and at the seafloor. Reasonable people can disagree on whether dispersants should have been used given the absence of any real preparation for stopping a blowout. I was very critical of dispersants as a response, partly because they exemplified the lack of real preparation, their chemical components were being kept secret, and they served BP's interests in hampering understanding of the amount of oil leaking. Legal liability is based on how much oil enters the environment. This gives an oil company strong incentive to minimize or hide the amount of oil. Critics used the analogy of putting the perpetrator in charge of securing and cleaning up a crime scene. (BP's liability assessment will largely be based on how much oil leaked. BP's first estimate of the amount of oil leaking was 1,000 barrels/d, one-sixtieth the amount—roughly 60,000 barrels/d—later determined and used by the US Coast Guard [1 barrel = 42 US gallons, ≈160 liters]. In December 2010, BP announced it would contest the official and academic estimates of the total amount of oil.).

Although minor blowouts are not uncommon, and more serious ones occur periodically, plans for responding to a blowout were essentially nonexistent. Drilling technology had improved radically, but response technology and preparedness had not changed in decades. The various caps that were tried and failed to stop the 2010 blowout were similar to those that failed in 1979 to stop the Ixtoc blowout, which leaked 140 million gallons of oil into the Gulf of Mexico over 9 months. The device that eventually stopped the Macondo blowout was designed and built specifically for that purpose; critics likened it to responding to a burning building by designing and building a fire truck.

US oil spill response law, enacted in 1990 to prevent another *Exxon Valdez*–like tanker surface spill, was likewise unprepared for a blowout in deep water. The law required the spiller to conduct the cleanup, often putting the Coast Guard in a position of seeming to serve rather than direct the response. This situation also let BP inject money into expensive projects that politicians wanted, but that experts criticized as ineffective, ecologically damaging, and a waste of money. I witnessed wetlands being destroyed in Dauphin Island, Alabama, to harden shorelines and dig sand for miles of berms that looked like they would wash away in the first substantial wind.

## How Bad Is It?

Mid-summer assurances by some federal officials that the oil was “gone” [2] angered regional residents and cost federal agencies the public's trust. Some residents, business owners, fishing interests, environmentalists, and media fueled panicky predictions of permanent ruination of beaches and fisheries, crop failures from oil lifted by hurricane winds and swept inland, and even—in one widely circulated email—nothing less than death of the entire planet from a massive methane release that the blowout would trigger.

Nor were scientists blameless in making overstated claims. Some scientists predicted scenarios that were very unlikely, such as thick oil blanketing the East Coast after getting entrained in the Loop Current and Gulf Stream.

The *Exxon Valdez* was most people's mental picture of likely damage. In that case several factors, including the enormous numbers of birds and mammals killed and Exxon's callous response to the local community, had indeed added up to a catastrophe causing serious long-term ecological, economic, and psychological damage.

Widespread predictions notwithstanding, the long-term effects will not be known until the long term. How long, where, and with what effect oil will remain in marsh and seafloor sediments also remains to be seen. The effects of oil on plankton, larval fish, many invertebrates, food webs, and other ecological interactions are largely unknown, and may never be known [3].

Oil has been found on the seabed in some areas and appears to have killed some benthic infauna and deep sea corals [4]–[7]. Hundreds of birds were brought to rehab facilities, indicating likely uncollected mortalities in the low thousands. (*Exxon Valdez*, though it spilled far less oil, killed an estimated quarter million birds and thousands of otters and seals. The physical configuration of that spill and semi-enclosed nature of Prince William Sound, the viscosity of the crude, the cold, and the presence of different families, orders, and densities of birds and mammals contributed to that difference [1].) Effects on fish await further monitoring, but fishing bans greatly reduced fishing mortality, and when areas closed because of oil were reopened, fishing was reportedly excellent. Oil appeared to kill some adult dolphins, but there seem to be no well-documented mass kills. I saw dolphins swimming in moderate oil; perhaps many evaded heavy oil. The cause of recent elevated mortality of newborn dolphins—not unprecedented—remains to be evaluated. The critically endangered Kemp's ridley turtle was likely the Gulf species most vulnerable to extensive surface oil. Whether their recovery has suffered a measurable setback remains to be seen. Migrations put many sea turtles in the open Atlantic during the summer, away from the oil. About 500 turtles were found debilitated or dead during the blowout, but a significant number showed no obvious signs of oil, suggesting that other causes—possibly including the nets of shrimp fishers hurrying to maximize landings prior to inevitable shut-downs—may have contributed. Some 70,000 turtle eggs were transplanted to Florida's east coast, which may boost those nesting populations, but will cost Gulf turtle populations the great majority of this year's cohort. Understanding the effects on adult turtle numbers will have to wait years, as future breeding seasons inform researchers of the effects of this year's mortality on adults and future maturing juveniles (sea turtles take roughly 12 to 20 years to mature, and do not breed annually).

## Relative Harms

Many people, including the President of the United States, said that the 2010 Deepwater Horizon disaster—which was the largest unintended release of petroleum ever—constituted “the worst environmental catastrophe in American history.” (Some simply said “in history.”)

But “worst” compared to what? The blowout's main effects already seem temporary compared to the clear-cutting of most of the Pacific Northwest's forests, conversion of prairies, disappearance of once-vast populations of wildlife including the now-extinct passenger pigeon and Eskimo curlew, near-extermination of vast herds of bison, deep depletion of formerly teeming fishes like Atlantic cod and bluefin tuna, and so many other long-term alterations of abundance and distribution. The most recent academic expert opinion predicts most commercially exploited Gulf marine resources except Louisiana oysters (many of which were killed by freshwater diverted in an attempt to push oil away from the coast) will return to pre-blowout abundances this year [3].

On a global scale, the blowout appears small and fleeting in comparison to deforestation, accelerating species loss, freshwater depletion, fisheries collapses, human population expansion, polar melting, coral bleaching, and changes to the planet's heat balance and the seas' chemistry.

Beyond the US, spilled oil routinely causes worse problems for nature and people than did the 2010 Gulf blowout. Nigerians could scarcely believe the efforts exerted to stop the Gulf oil leak and to protect the Gulf shoreline, because an amount of oil roughly equivalent to the *Exxon Valdez* spills into the Niger Delta every year [8]. Such spills have destroyed farms and forests, contaminated drinking water, driven people from their homes, and ruined the nets and traps of fishing people. In the first week of May 2010, a ruptured ExxonMobil pipeline spilled more than a million gallons into the Niger Delta over seven days. Oil companies claim these leaks are caused by vandals. Many Nigerians blame rusting facilities, and believe that oil companies simply don't care. A member of Nigeria's parliament commented, “Oil companies do not value our life; they want us to all die.” The Nigerian government estimated that more than 200 spills per year occurred annually between 1970 and 2000.

In 2009, poor safety procedures and avoidable human error on the Thai-owned West Atlas drilling rig caused a blowout in the Montara field in Australian waters. It leaked an estimated 30,000 barrels of oil that drifted over 90,000 km^2^ to Indonesian waters over 74 days before a relief well plugged it. The Australian government's report said the rig was “an accident waiting to happen; the company's systems and processes were so deficient and its key personnel so lacking in basic competence, that the blowout can properly be said to have been an event waiting to occur. Indeed…the Inquiry discovered that not one of the five Montara wells currently complies with the company's Well Construction Standards” [9]. Indonesia demanded US$2.4 billion in compensation, which the oil company rejected [10].

## Mounting Crises

At a Washington, D.C., hearing in the spring of 2010, a US congressman wept because he could not bear the thought that the oil might destroy the wetlands of the Mississippi Delta. For much of the summer, that fear was widely shared.

But the Associated Press calculated that, along the Louisiana coastline and roughly 18,000 km^2^ (7,000 mi^2^) of marsh, only 9 km^2^ (3.4 mi^2^) of marshland got oiled. Some of that vegetation was already re-growing by late summer.

Yet while oil touched the fringes, water diversions, flood control, and 16,000 km (10,000 mi) of channels cut into the Mississippi River Delta have done—and continue doing—real and permanent damage.

The great Delta of the Mississippi once spanned roughly 22,000 to 25,000 km^2^ (8,500 to 10,000 mi^2^). About 20 percent of that area, or very roughly 5,000 km^2^ (1,800 mi^2^) of marsh, has vanished. Recent loss rates have been estimated around 100 to 200 km^2^ (20 to 40 mi^2^) a year. All these estimates vary, as do the reasons. Hurricanes Katrina and Rita dissolved over 200 square miles of marsh to open water, but that is because much of that marsh had already been degraded.

The Mississippi River is the main source of sediment that is the Delta's nourishment, but engineering projects have almost completely isolated the river from its delta. Because of levees built to control floods, and the thousands of miles of channels sliced through the marshes for shipping and for vessels servicing the oil rigs, sediment that might build the marsh goes straight out to the open Gulf. Oil and gas pumping have also helped the marshes subside. Sea level rise adds to the problem. The marshes are a major reason that the Gulf produces more seafood than elsewhere in the lower 48 states. But expect continued loss of marshes and wet cypress forest, affecting wildlife, recreation, and fisheries productivity: some estimates predict the marshes will largely vanish by 2050 [11],[12]. These problems existed before the 2010 blowout, and continue afterwards. They have destroyed more marsh than the 2010 blowout, and they continue to drive the region's worst environmental calamity.

Was the largest accidental release of petroleum in history really even the worst “spill”? In the blowout, surface oil affected roughly 5 percent of the entire Gulf [3]. Approximately 200 million Macondo gallons hemorrhaged into the Gulf's 660 quadrillion gallons of water. That volume of water would dilute the oil, and the Gulf's microbes would begin metabolizing it. But the carbon dioxide we're spilling into the atmosphere isn't getting diluted; it's becoming more concentrated. The greatest environmental catastrophe is not the oil we spill. It's the oil and coal we burn in our engines, whose resulting carbon dioxide is altering the heat balance of the planet and the acidity of the seas. It is melting polar systems, dissolving shellfish, killing coral reefs, and changing the abundance and distribution of organisms in many systems worldwide. Combustion, not leakage, is the real disaster. And even combustion would be far less a problem if not for the sheer force of our overwhelming numbers.

## Lessons and Implications

One lesson from the events that caused the blowout is that human judgment is too frail and self-filtered to prevent all future accidents associated with deep drilling. Because deep-water accidents are difficult to contain, the stakes are increasingly high, with broad potential effects to natural assets that support regional economics like fishing and tourism. Much stronger government oversight could help. Drillers are turning to deep water because easier, less expensive, shallower, and land-based reservoirs are being depleted. Increasing the complexity of the operation will always increase the risk.

The Obama Administration has reversed its plan to expand offshore drilling. But the oil industry will continue to push for more drilling in more places. The country will not simply say no to new drilling, so we must also say yes to fast-tracking the scale-up of clean energy technologies.

We may have dodged a bullet with the Deepwater Horizon blowout because the water is warm, the crude is light, the microbes are hungry, and the walruses are far to the north. But it was, nonetheless, a multi-billion-dollar trauma to Gulf coast communities and businesses.

If the same thing had happened where walruses really do live—where many people want to drill, baby, drill—the damage would be much greater and longer-lasting, with deeper ecological effects, and a vastly slower, more difficult response as a result of the distances between major human population centers and ports. And the natural response would not benefit from the Gulf of Mexico's unique “long term tolerance and adaptation to chronic additions of hydrocarbons” conditioned by microbes adapted to the Gulf's roughly 1,000 natural petroleum seeps [3]. The Gulf of Mexico was arguably the best place in the world for such a large accidental release of petroleum. It likely represents the best case, not the mean. Next time, we may not be so “fortunate.”

In 1969 the Santa Barbara, California, oil blowout shocked the nation and helped create the pivotal impetus for the burst of bipartisan environmental legislation of the 1970s. The main value that might have come of the Deepwater Horizon blowout would have been in creating game-changing momentum toward a new energy path. But the country's current political polarity and a bitterly partisan Congress helped prevent that from happening. It appears the moment was lost. Without that moment spurring policies encouraging stepped-up development of new energy options, the blowout was just a mess with no redeeming value. Its instructiveness has not been fully assimilated.

The US needs the new investment, construction, and infrastructure for phasing in a diversified cleaner energy future. But as long as Big Oil and Big Coal continue to get market-distorting subsidies, and as long as elections are undermined by corporate money, building the needed energy future will be very difficult for the US to achieve. The main problems existed before the oil blowout. And afterward, they remain.

This does not suggest that the risks of deeper-water drilling are trivial. Brazil's president has called its recent deep-water oil discoveries “a gift from God.” But Claudio Sampaio of the University of São Paulo observes, “We are talking about a complex and aggressive environment: there's salt, there's corrosion, extreme pressures, weather can change, waves of 10 m (33 ft) can appear from nowhere…There's no engineering solution that could be 100% safe” [13]. Brazil's Tupi field lies 300 km (≈190 miles) offshore, in water 2 km (1.3 mi) deep, under 5 km (≈16,000 ft) of sand, rock, and thick, rock-hard salt [14]. Indeed, as drilling goes deeper—especially where oil companies operate with insufficient oversight and relative impunity as in the Montara example—the risks to regional coasts, marine life, fisheries, reefs, and poor peoples increases. The 2010 Deepwater Horizon blowout was yet another warning that the world's future energy needs cannot be met by sources that cause such large acute and chronic problems and are getting more difficult and more complicated to extract.
